# Letrozole, an aromatase inhibitor, improves seminal parameters and hormonal profile in aged endangered Markhoz bucks

**DOI:** 10.5713/ab.21.0271

**Published:** 2021-08-25

**Authors:** Ako Rezaei, Asaad Vaziry, Abbas Farshad

**Affiliations:** 1 Department of Animal Science, Faculty of Agriculture, University of Kurdistan, Sanandaj 661715175, Iran; 2 Department of Animal Science, Kurdistan Agricultural and Natural Resources Research and Education Center, AREEO, Sanandaj, 6616936311, Iran

**Keywords:** Aged Bucks, Aromatase Inhibitors, Seminal Plasma, Sex Hormones, Testis

## Abstract

**Objective:**

Letrozole, a potent aromatase inhibitor, is known to have the potential to modify male reproductive function by altering sex hormone levels. This study aimed to evaluate the semen and testicular characteristics and hormonal profile of aged Mrakhoz bucks (*Capra hircus*) treated with letrozole.

**Methods:**

Twelve Markhoz male goats, aged between 4.5 to 5.5 years with an average body weight (BW) of 61.05±4.97 kg were used for the study. Animals were randomly divided into two equal groups and subcutaneously received either 0.25 mg/kg BW of letrozole or a control every week for 2 months. The semen collections were performed every 10 days, and blood samples and testicular biometric records were collected at 20 days intervals.

**Results:**

Letrozole causes increased testosterone and follicle-stimulating hormone levels, testosterone to estradiol ratio, semen index and reaction time during the period from 20th to 60th days (p<0.05). Furthermore, letrozole-treated bucks had higher semen volume, sperm concentration, and total sperm per ejaculate from 30th to 60th days (p<0.05). However, no differences occurred between the groups in scrotal circumference, relative testicular volume, semen pH, abnormality, acrosome integrity, and membrane integrity of sperm during the study (p>0.05). The serum luteinizing hormone levels, sperm viability, motility, and progressive motility increased, and estradiol levels decreased after 40th to 60th days of letrozole treatment (p<0.05).

**Conclusion:**

Letrozole application to aged Markhoz bucks provokes reproductive hormonal axis which, in turn, induces enhancement of semen production and quality.

## INTRODUCTION

Markhoz goat is an endangered species in Iran and one of the two breeds of mohair producers in the world. In the last decade, the geographic distribution of Markhoz goats has severely been diminished especially in its main native area in the Kurdistan province. Most of the remaining population is being bred at two husbandry research stations [1]. The integration of *in situ* and *ex situ* programs aimed at maintenance viable populations and germplasm cryopreservation has been documented as a principal approach in setting national conservation priorities of Markhoz goats. The aging-related decline in fertility could be one of the factors in the reduction in buck population size in the breeding pool. The conservation of such genetically superior bucks is valuable for the transmission of unique genetic factors to offspring. Studies have shown that the optimal reproductive status in mature bucks decreased from the age of 4 to 5 years onwards, with a decline in testosterone levels [2]. In Markhoz bucks, the older (aged 4.5 to 5.5 years) had lower semen quality and quantity than the younger (aged 2.5 to 3.5 years) (data not shown). Achievement of complete spermatogenic potential for a successful reproductive outcome depends on higher levels of testosterone in seasonally breeding animals [3]. The age-related reduction of testosterone synthesis is probably related to the luteinizing hormone (LH) -suppressive activity on Leydig cell steroidogenic capacity [4]. Hence, any promotion in gonadotropin-dependent testosterone secretion may contribute to successful fertility preservation of aged endangered animals [5].

There Aromatase inhibitors act as hormone-targeting agents that have the potential to interfere with the male reproductive function by altering the sex hormone levels towards testosterone. Letrozole, as a non-steroidal class of third-generation aromatase inhibitors, is comprised of the 1, 2, 4-triazole nucleus which reversibly binds to the heme groups of aromatase enzymes mainly located in testicular, hepatic and brain tissues resulting in estrogen synthesis reduction [6]. This estradiol reduction can increase testosterone levels directly by accumulating testosterone that has not been converted to estrogen and/or indirectly by promoting gonadotropin secretion via the negative feedback on the hypothalamic-pituitary axis, which results in improved male fertility [7].

In men, the restorative effects of letrozole on sex hormonal profile and/or spermatogenesis have been confirmed in conditions associated with low testosterone levels such as aging [8], obesity [9], nonobstructive azoospermia [10], idiopathic oligo/astheno/teratozoospermia [11], and other fertility disorders [12]. Furthermore, a number of male animal studies have shown that a positive relationship exists between inhibition of aromatase and reproductive performance [13–15].

In recent years, aromatase inhibitors are highly regarded for their efficient performance on age-related infertility in the rooster [16]. Letrozole therapy has been proposed as a useful strategy that resulted in improved sperm quality, gonadotropins, and testosterone in aged-roosters [17–19]. In our previous study, we found that letrozole improved hormonal profile, testicular and seminal characteristics during the puberty of Markhoz bucks [20], but whether this aromatase inhibitor has positive reproductive effects on these endangered goats in older ages remains unknown. Thus, the objectives of present study were to evaluate the effects of letrozole on semen characteristics and hormonal profile in aged Markhoz bucks.

## MATERIALS AND METHODS

### Animal care

Ethical considerations and animal maintenance were approved by Research Ethics Committee of University of Kurdistan (IR.UOK.REC.1398.022).

### Animal and treatments

**Figure 1:**
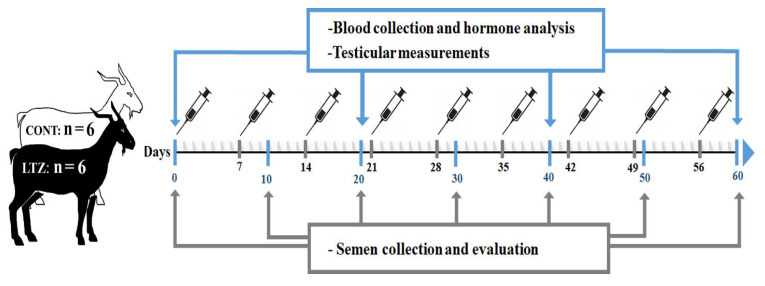
Diagrammatic representation of the experimental design, and sampling schedule in aged Markhoz bucks treated letrozole (LTZ) (0.25 mg/kg BW/wk) or control (CONT).

A diagrammatic representation of the experimental process is schematized in Figure 1. Twelve Markhoz bucks aged between 4.5 to 5.5 years, and average body weight (BW) of 61.05±4.97 kg were assigned to the study. Animals were kept from 8 October to 8 December (the physiological breeding season) in the Sanandaj Animal Husbandry Research Station (Kurdistan province, longitude 46.99°E, latitude 35.31°N). The clinical health status and external genitalia conditions were all normal for the entire study period. They were maintained optimally under uniform nutritional conditions and exposed to the natural photoperiod. Bucks were randomly divided into two groups (n = 6 per group) and subcutaneously received either 0.25 mg/kg BW/wk of letrozole (Pharmaceuticals Co, Tehran, Iran) or a control for 60 consecutive days. The letrozole treatment was prepared using 0.3% hydroxypropylcellulose (Sigma-Aldrich, St. Louis, MO, USA) in 0.9% NaCl solution with letrozole to obtain the required concentration. The control was prepared using 0.3% hydroxypropylcellulose in 0.9% NaCl solution without letrozole. The resulting injection volumes for each buck was 2 mL.

### Testicular measurements

Relative testicular volume and scrotal circumference were measured at 20 days interval through experimental period. Tape measure graduated in mm was used to determine scrotal circumference. Testicular (scrotal sac) volume was estimated using the volume of water dislodged on the measuring cylinder method according to Archimedes law of buoyancy. Relative testicular volume was calculated as testicular (scrotal sac) volume divided by BW [21].

### Blood collection and hormone analysis

Blood samples in the morning (between 8:00 and 9:00 am) after an overnight fast were drawn at 20 days interval via the jugular vein into non-anticoagulant tubes. The blood was then centrifuged at 3,000 rpm for 20 min, and the sera were collected and stored immediately at −20°C until assayed. The commercially available AccuBind kits (Monobind Inc., Lake Forest, CA, USA) were used to quantify serum testosterone, estradiol, LH, and follicle-stimulating hormone (FSH) levels based on the manufacturer instructions, by enzyme-linked immunosorbent assay (ELISA) microplate reader with Gen5 Software (ELx808, Biotek Instruments, Winooski, VT, USA). The intra- and inter-assay coefficients of variation were 6.4, 9.8% for testosterone, 7.2, 10.7% estradiol for estradiol, 7.5, 11.4 for LH and 4.5 and 5.3% for FSH assay. The sensitivity of the assay was 0.06 ng/mL, 10 pg/mL, 0.054 mIU/mL and 0.134 mIU/mL for testosterone, estradiol, LH and FSH, respectively.

### Semen collection and evaluation

Semen was obtained early in the morning at 10-day intervals by using an electro-ejaculator (Lane Manufacturing Inc., Denver, CO, USA). Reaction time as the elapsed time between the onset of the electro-stimulation and ejaculation was recorded using a stopwatch at each semen collection [22]. The ejaculates were poured into pre-warmed graduated tubes maintained at 37°C and its volume recorded to the nearest 0.1 mL. Digital pH-meter (Hanna pH 211; Hanna Instruments, Padova, Italy) was used to measure the seminal pH. Sperm concentration was evaluated after dilution and staining with eosin using a hemocytometer according to Farshad et al [23]. The total number of sperm per ejaculate was then calculated (semen volume×sperm concentration). For the assessment of sperm motility (total and progressive), semen sample diluted with 0.1 M sodium citrate solution (5 μL) was placed on a pre-warmed glass slide, covered with a coverslip, and examined by counting a minimum of 200 sperm using a phase-contrast microscope at 200× magnification. The motility parameters of each sample were the average of five observer records. Eosin–nigrosin stain procedure was performed to assess sperm viability and total abnormality [23]. Briefly, sperm smears were prepared by mixing a 10 μL of diluted semen with 10 μL eosin-nigrosin stain on a warm slide and immediately spreading the mixture with a second slide. After air drying, a total of 200 sperm were counted using a phase contrast microscope and classified as either non-stained (viable) or stained (dead). Semen index, as a semen quality indicator, was calculated based on the method described by Talebi et al [3]. The percentage of acrosomal integrity was evaluated in formol citrate-fixed samples. In brief, 50 μL of semen sample was mixed with 500 μL of 1% formal citrate (2.79% tri-sodium citrate dehydrate and 0.37% formaldehyde in distilled water), smeared onto a glass slide and air-dried. At least 200 sperm were counted under 1,000× magnification to assess intact acrosome that showed normal apical ridge. For assessment of sperm membrane integrity, 30 μL of semen with 300 μL of a 100 mOsm hypo-osmotic solution (9 g fructose + 4.9 g sodium citrate per liter of distilled water) was incubated at 37°C for 60 min. This mixture (0.2 mL) was spread with a cover slip on a warm slide. A total of 200 sperm was counted under 1,000× magnification in at least five different microscopic fields. The percentage of sperm that swelled under hypotonic conditions was recorded [20].

### Statistical analysis

Before conducting analysis, data were checked for normality and homogeneity of variance by the Shapiro Wilk and Levene’s test, respectively. Data were statically analyzed under a completely randomized design with repeated measurements using the MIXED procedure of SAS version 9.1 (SAS Institute Inc., Cary, NC, USA). This model included fixed effects of treatment, time, and treatment×interaction. The statistical significance level for all analyses was defined as p<0.05.

## RESULTS

### Testicular and semen characteristics

The results in Table 1 indicate that scrotal circumference and relative testicular volume did not differ between the groups during the experimental period. The effects of letrozole on the semen characteristics of Markhoz bucks are summarized in Table 2. Letrozole significantly improved semen volume, total sperm per ejaculate and concentration after 30 to 60 days of treatment (p<0.05). Sperm viability, motility, and progressive motility were higher (p<0.05) in the letrozole group than in the control during the 40, 50, and 60 days of the experiment. Furthermore, bucks treated with letrozole showed a marked increase in reaction time and semen index from day 20 until the end of the experiment as compared with the control group (p<0.05). Nevertheless, no change occurred in the semen pH, abnormality, acrosome integrity, and membrane integrity of sperm between the treatment and control bucks during the study (p>0.05).

### Hormonal levels

**Figure 2:**
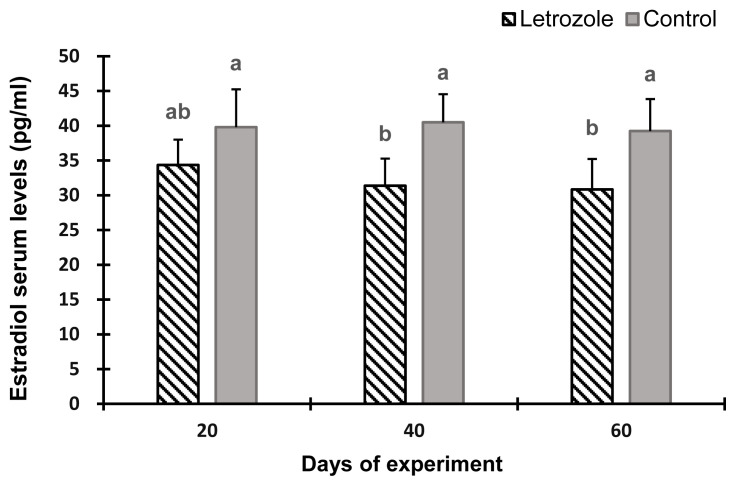
Estradiol levels in Markhoz bucks treated letrozole (0.25 mg/kg BW/wk) or control. Error bars indicate standard deviation. ^a,b^ Statistically significant differences (p<0.05) between the groups.

**Figure 3:**
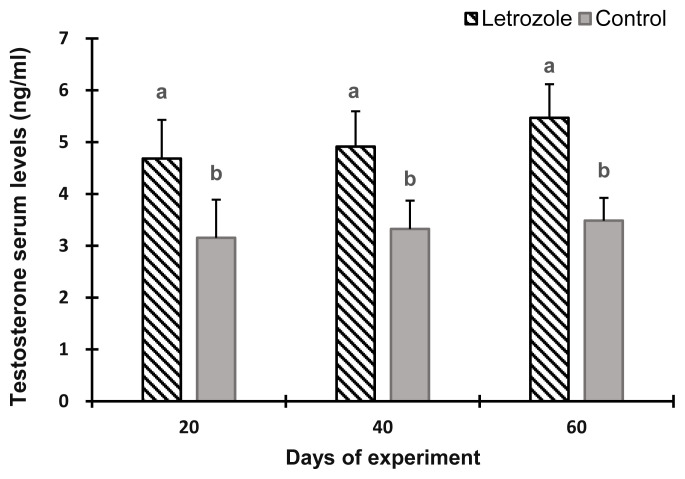
Testosterone levels in Markhoz bucks treated letrozole (0.25 mg/kg BW/wk) or control. Error bars indicate standard deviation. ^a,b^ Statistically significant differences (p<0.05) between the groups.

**Figure 4:**
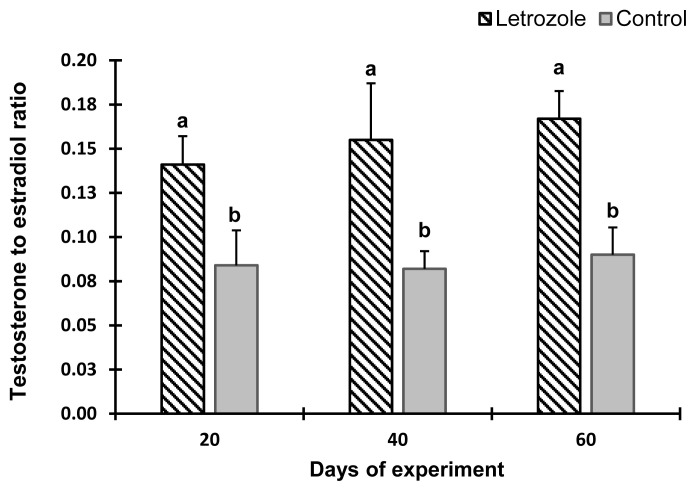
Testosterone to estradiol ratio in Markhoz bucks treated letrozole (0.25 mg/kg BW/wk) or control. Error bars indicate standard deviation. ^a,b^ Statistically significant differences (p<0.05) between the groups.

**Figure 5:**
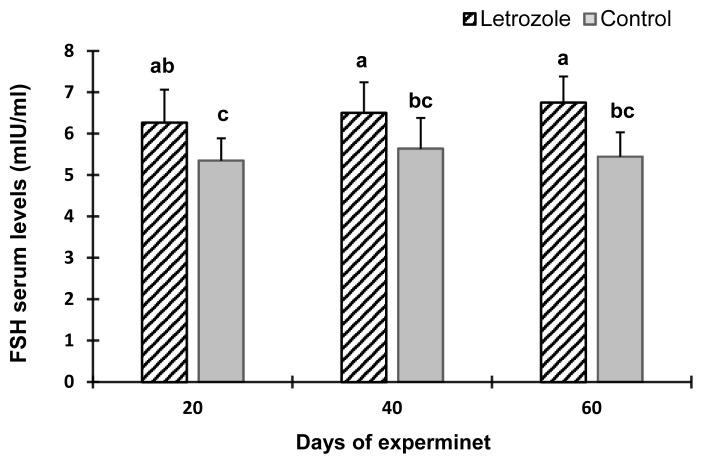
Follicle-stimulating hormone (FSH) levels in Markhoz bucks treated letrozole (0.25 mg/kg BW/wk) or control. Error bars indicate standard deviation. ^a,b^ Statistically significant differences (p<0.05) between the groups.

**Figure 6:**
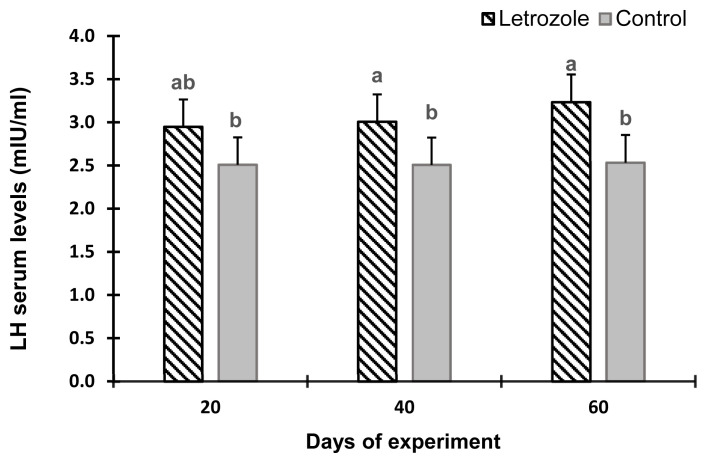
Luteinizing hormone (LH) levels in Markhoz bucks treated letrozole (0.25 mg/kg BW/wk) or control. Error bars indicate standard deviation. ^a,b^ Statistically significant differences (p<0.05) between the groups.

The serum estradiol levels of the letrozole group were lower (p<0.05) at 40 and 60 days of the experiment compared to the control group (Figure 2). In contrast, the serum testosterone levels (Figure 3), testosterone to estradiol ratio (Figure 4), and serum FSH levels (Figure 5) increased significantly from 20 to 60 days of the experiment in letrozole treated animals as compared to the control group (p<0.05). In addition, notable increases (p<0.05) in serum LH levels were found in the letrozole group as compared to the control group during days 40 and 60 (Figure 6).

## DISCUSSION

The male peripheral aromatase activity enhances with advanced age, resulting in increased estrogen synthesis from androgen which may lead to lower testosterone levels [6]. Aromatase inhibitors have been used in the last decade in an attempt to ameliorate fertility in older men [7,12] and roosters [16–19]; hence, it is likely that suppression of aromatase favorably affects reproductive efficiency in other aged animal species. In support of this hypothesis, we observed for the first time that letrozole, as an effective aromatase inhibitor, markedly improved semen quality and quantity of aged bucks associated with augmented testosterone to estradiol ratio, and serum levels of testosterone and gonadotropins. These changes in hormone levels are in agreement with those of Dias et al [7] who found that aromatase inhibitor-induced declined estradiol levels were associated with elevated serum FSH and LH levels and consequently increased circulating testosterone in aging men. The major source of aromatase substrate, a prerequisite for estrogen production, has been shown to exist in the testicular tissues, mainly Leydig, Sertoli and germinal cells. In men [6], rat [24], and ruminants [20], estrogen acts as a potent negative feedback modulator on the hypothalamus-pituitary-testicular axis and can alter the GnRH and gonadotropin secretion. In males of these species, decreasing estrogen levels by aromatase inhibitors can stimulate GnRH secretion, which in turn, increases the FSH and LH levels, and simultaneously, these high LH levels stimulate intratesticular testosterone production via the Leydig cells. Hence, hormonal change strategy by aromatase inhibitors has been considered for maximal testosterone output in aging males. As gerontology studies have shown, age-dependent changes in sex hormones result in a reduction in the ratio of testosterone to estradiol levels, which negatively affects fertility [6,7]. Therefore, it seems that letrozole administration modifies the serum hormonal profile of LH/testosterone and FSH levels by decreasing the estradiol levels, which can improve variables associated with the reproductive success of aged Markhoz bucks.

In the present study, significantly higher semen index, semen volume, sperm concentration, total sperm per ejaculate, sperm viability, motility, and progressive motility were observed in letrozole-injected aged bucks. This is consistent with many previous studies [6,10–12] which have demonstrated that aromatase inhibitors improved the sperm parameters in ejaculates of men with infertility problems by increasing the testosterone/estradiol ratio. In similar study, Turner et al [24] asserted that spermatogenesis efficiency increased by treating the adult rats with anastrozole, as an aromatase inhibitor. In line with our results, Stein et al [13] has demonstrated that the elevated volume of ejaculates in stallion were associated with enhanced serum testosterone levels after 60 days of letrozole administration. Earlier studies on boars indicated that the oral letrozole treatment improved sperm production capacity through stimulating Sertoli cell proliferation in response to estrogen deficiency [14,15]. Nevertheless, a report in calves by Berger [25] showed that although oral letrozole was associated with lower estradiol, it did not affect testosterone levels and Sertoli cell number, which might be due to the incomplete absorption of letrozole by the ruminal route and other factors such as age and species. We have previously shown that the Sertoli cell number increased in the testis of the letrozole-injected bucklings [20], as also earlier shown in boars. Sertoli cells, as the main testicular target cell for FSH and testosterone, provide a permissive environment that facilitates differentiation, transformation, and replication of germ cells to spermatozoa is via direct contact [15]. The nectin-3 protein is mainly expressed in Sertoli cells within the testis and plays an important role in the spermatogenesis process via Sertoli-spermatid junctions. In this regard, letrozole administration increased expression of the nectin-3 gene associated with improved the hormonal status and fertility of aged roosters [19]. Based on the above-mentioned evidence, letrozole can stimulate Sertoli cells by i) increasing LH-induced testosterone secretion; ii) directly increasing FSH levels; iii) reducing estrogens or/and receptor estrogen blockade. Therefore, the improved sperm parameters in our letrozole-treated bucks may partly reflect the Sertoli cell response to hormonal modification. However, there is a need for further research on this topic.

Another interesting finding is that aromatase inhibitors improved the mitochondrial activity of rooster sperm which was associated with better motion characteristics [16]. The largest fraction of total ATP production in sperm mitochondria consumed by motility, hence, the amount of metabolic energy sources is mainly linked to sperm movement. Aging is associated with reduced circulatory testosterone production and testicular glucose metabolism. An *in vitro* study on rats conducted by Banerjee et al [26] showed that LH treatment increased glucose, lactate and GLUT 8 protein levels in testis by increasing testosterone output. Similarly, Ali et al [18] concluded that the reason for improved sperm quality observed in aged roosters treated with letrozole is possibly due to an increase in sperm energy production, which has been shown to increase with stimulation of LH-induced testosterone secretion. This agrees with our data that showed improved sperm motility and progressive motility were accompanied by increased LH levels after 40 days of letrozole treatment in aged bucks.

On the other hand, the effect of FSH application in reducing sperm DNA damage by promoting anti-apoptotic and maturation on testicular cells is documented. The DNA fragmentation index is closely correlated with sperm parameters, especially, sperm morphology and count. FSH is probably involved in the replacement of histones with protamines and reduced DNA fragmentation during this process that may lead to improved sperm maturation [27]. In the present study, a significant increase in sperm concentration and total sperm per ejaculate as well as a slight tendency to decrease in sperm abnormality after letrozole treatment could be related to the high FSH levels in aged bucks. Congruent with this, Kooshesh et al [11] indicated that letrozole administration acts as a fertility agent in idiopathic infertile men, reducing protamine deficiency and DNA fragmentation affected by chromatin packaging and apoptosis pathways by stimulating FSH levels.

High-volume ejaculates of letrozole-treated bucks may be related to more activities of the epididymis and accessory glands in response to increased testosterone secretion in this study. The accessory glands are considered as androgen-dependent tissues and their secretions constitute the largest volume of seminal plasma. The presence of aromatase has been detected in epididymis, seminal, and prostate glands in the goat [28]. Therefore, the inhibition of aromatase can increase local androgen not converted to estrogen, which may improve the reproductive tract secretions such as biochemical components and other vesicular products into the seminal fluid [20].

The shorten reaction time accompanied by increased testosterone levels after letrozole treatment in this study partially agrees with an observation by Ángel-García et al [29], who found that increase serum testosterone levels positively affected the reaction time in male goats. Testosterone induces sexual behavior activation by binding to androgen receptors which act on the central nervous system. Similarly, a study on bucks indicated that the testosterone application clearly provoked sexual activity by increased frequency of courtship, mount, ejaculation, and self-enurination [30]. Thus, further research is required to evaluate more specifically the letrozole effects on sexual behavior activities in bucks.

In the present study, testicular biometric parameters were not significantly affected by letrozole administration. The reasons why these variables were not affected by the letrozole, are not fully elucidated, but this may be related to the aging of experimental male goats that is consistent with the report on aged roosters by Adeldust et al [17] who did not observe any changes in testicular index after letrozole treatment. In ruminants, age-related increases in testicular degeneration are associated with a decrease in scrotal circumference [2]. Hence, according to the positive results of sperm quality in our letrozole-treated animals, the stability of testicular weight and volume during aging can be considered favorable. Based on our results, we suggest that letrozole treatment as an alternative method to stimulate transiently fecundity activity through the efficient production of high-quality semen that may be useful for use in mating and/or artificial insemination of older purebred Markhoz bucks.

In conclusion, letrozole administration in weekly intervals for 2 months was effective in stimulating the reproductive axis of aged bucks. During most of the experimental period, the semen production and quality were improved by letrozole treatment. It appears that the letrozole-induced stimulatory effect on testicular function is mediated by modified levels of LH/testosterone and FSH in response to reduced estradiol levels; this can contribute to the reproductive success in older Markhoz bucks.

## Figures and Tables

**Table 1:** Testicular biometric parameters in Markhoz bucks treated letrozole (0.25 mg/kg BW/wk) or control

Parameters	Days of experiment (d)			SEM	p-value

	20	40	60		
Relative testis volume (cm^3^/kg/BW)					
Letrozole	6.89	6.97	7.02	0.064	0.697
Control	6.84	6.87	6.93		
Scrotal circumference (cm)					
Letrozole	27.58	27.84	27.80	0.203	0.292
Control	26.92	26.96	27.06		

BW, body weight; SEM, standard error of mean.

**Table 2:** Semen characteristics in Markhoz bucks treated letrozole (0.25 mg/kg BW/wk) or control

Parameters	Days of experiment (d)						SEM	p-value

	10	20	30	40	50	60		
Reaction time (s)								
Letrozole	35.2^bc^	33.2^c^	31.4^d^	29.0^d^	29.8^d^	28.2^d^	0.833	<0.0001
Control	39.1^c^	41.8^a^	40.6^ab^	38.6^abc^	42.2^a^	41.0^a^		
pH								
Letrozole	6.94	6.96	7.04	6.96	7.00	6.92	0.034	0.783
Control	7.01	6.97	7.03	6.92	6.93	6.90		
Volume (mL)								
Letrozole	0.70^abcd^	0.74^abcd^	0.80^abc^	0.82^ab^	0.83^a^	0.82^abc^	0.017	<0.0001
Control	0.66^cd^	0.62^d^	0.64^d^	0.66^cd^	0.64^d^	0.66^cd^		
Sperm concentration (×10^9^/mL)								
Letrozole	2.98^abc^	3.04^abc^	3.17^ab^	3.21^a^	3.22^a^	3.29^a^	0.038	<0.0001
Control	2.82^c^	2.79^c^	2.80^c^	2.84^bc^	2.87^bc^	2.85^bc^		
Total sperm (×10^9^)								
Letrozole	2.08^bcd^	2.26^ab^	2.53^ab^	2.66^a^	2.67^a^	2.71^a^	0.067	<0.0001
Control	1.80^cd^	1.73^d^	1.78^cd^	1.94^cd^	1.83^cd^	1.87^cd^		
Viability (%)								
Letrozole	65.2^abc^	66.4^abc^	67.0^abc^	67.8^ab^	68.6^a^	67.8^ab^	0.636	<0.0001
Control	62.6^bc^	61.6^c^	62.4^bc^	62.0^c^	62.2^c^	61.6^c^		
Abnormality (%)								
Letrozole	25.2	23.4	23.8	25	23.4	24	0.299	0.084
Control	25.6	24.8	25.6	26.2	25.4	25.8		
Motility (%)								
Letrozole	62.0^abc^	62.0^abc^	64.0^abc^	67.0^ab^	67.0^ab^	69.0^a^	1.004	<0.0005
Control	59.0^bc^	57.0^c^	61.0^abc^	58.0^c^	57.0^c^	60.0^bc^		
Progressive motility (%)								
Letrozole	57.0^abcd^	60.0^abc^	60.0^abc^	62.0^a^	61.0^ab^	63.0^a^	0.974	<0.0001
Control	53.0^bcd^	52.0^cd^	52.0^cd^	51.0^d^	51.0^d^	52.0^cd^		
Acrosome integrity (%)								
Letrozole	69.4	68.4	70.0	69.4	67.6	69.8	0.478	0.182
Control	69.0	67.6	68.6	68.2	65.2	67.8		
Membrane integrity (%)								
Letrozole	68.2	66.2	66.0	69.2	70.4	70.6	0.596	0.869
Control	69.80	67.4	67.2	68.0	68.6	68.2		
Semen index (×10^6^/mL)								
Letrozole	7691^cd^	9015^abc^	10,144^ab^	11,098^ab^	11,267^ab^	11,556^a^	360.1	<.0001
Control	6,017^d^	5,567^d^	5,853^d^	6,028^d^	7,214^cd^	6,031^d^		

BW, body weight; SEM, standard error of mean.

a–d Values with different superscripts in the same row are different (p<0.05).
